# Comprehensive identification and analysis of DELLA genes throughout the plant kingdom

**DOI:** 10.1186/s12870-020-02574-2

**Published:** 2020-08-06

**Authors:** Pengfei Wang, Qianqian Zhang, Yingchun Chen, Yanxia Zhao, Fengshan Ren, Hongmei Shi, Xinying Wu

**Affiliations:** 1 Shandong Academy of Grape, Shandong engineering research center for Grape cultivation and deep-processing, Jinan, 250100 China; 2 Key Laboratory of Urban Agriculture (East China), Ministry of Agriculture, Jinan, 250100 China

**Keywords:** DELLA, GRAS domain, PSG, NSG, Orthologous, Paralogous

## Abstract

**Background:**

DELLAs play key roles in plant gibberellin signaling pathways and are generally important in plant development and growth. However, DELLAs in many plant taxa have not yet been systematically analyzed.

**Results:**

In our study, we searched for DELLA genes across 58 green plant genomes and found 181 DELLAs. Structure analysis showed some DELLA domains do not contain “D-E-L-L-A” sequences and instead contain similar domains, including DGLLA and DSLLH domains. “VHYNP” motifs in plant DELLAs comprise 23 types of sequences, while some DELLAs did not contain GRAS domains. In grape, we found that the DELLA protein GSVIVT01015465001 contains an F-box domain, while apple DELLA proteins MDP0000220512 and MDP0000403162 contain a WW domain and a BCIP domain, respectively. These DELLAs can be divided into 22 homologous groups and 17 orthologous groups, and 35 paralogous genes were identified. In total, 35 positively selected genes (PSGs) and 121 negatively selected genes (NSGs) were found among DELLAs based on selective pressure analysis, with an average *K*_s_ of NSGs that was significantly higher than that of PSGs (*P* < 0.05). Among the paralogous groups, CBI and Fop were significantly positively correlated with GC, GC1, GC2, GC12, and GC3, while CAI was significantly positively correlated with GC, GC1, GC12, and GC. The paralogous groups with ω values exceeding 1 had significantly higher *K*_a_ values. We also found some paralogous groups with ω values exceeding 1 that differed in their motifs.

**Conclusions:**

This study provides helpful insights into the evolution of DELLA genes and offers exciting opportunities for the investigation of DELLA functions in different plants.

## Background

The development of a plant is an orderly process that starts from germination and continues to maturity, and it is modulated by environmental conditions and internal phytohormones, such as abscisic acid, cytokinins, ethylene, auxins, and gibberellin (GA). GA can modulate seed germination and stem and flower development processes, among other developmental processes [1–4]. *Arabidopsis thaliana* GA-deficient mutant *ga1–3* contains greatly reduced levels of endogenous GA; this mutant is defective in germination, impaired in the development of its flowers, and retarded in its vegetative growth [5–7]. DELLA proteins play a key role in the plant GA signaling pathway. DELLA proteins can regulate gene transcription, restrict plant development, and repress GA signaling [8, 9].

The most obvious feature of DELLA proteins is the N-terminal DELLA domain. Some DELLA domains (Pfam ID: PF12041) previously identified were found to contain “D-E-L-L-A” amino acid sequences [10]. DELLA proteins belong to the GRAS gene family and are a type of plant-specific nuclear protein. However, since DELLA proteins do not contain a canonical DNA-binding domain, they may interact with other transcription factors and then regulate the target genes. DELLA proteins can interact with many transcription factors, including ABA INSENSITIVE 3 (ABI3), ABI5, AUXIN RESPONSE FACTOR 6 (ARF6), PHYTOCHROME INTERACTING FACTORs (PIFs), BRASSINAZOLE-RESISTANT 1 (BZR1), ETHYLENE-INSENSITIVE 3 (EIN3), JASMONATE-ZIM-DOMAIN PROTEINs (JAZs), DWARF 14 (D14), and FLOWERING LOCUS C (FLC), as well as brassinosteroid (BR) signaling, which are involved in multiple phytohormone signaling pathways, thus participating in complex crosstalk among plant hormones [11–17].

In *A. thaliana*, five DELLA proteins were identified genome-wide: RGALIKE1 (RGL1), RGL2, RGL3, GA INSENSITIVE (GAI), and REPRESSOR OF GA1–3 (RGA). RGA, RGL1, and RGL2 have been shown to regulate floral development [18, 19]. RGL2 can also repress seed germination [20]. RGL3 represses testa rupture during seed germination [21]. RGA and GAI can repress vegetative growth [22, 23]. In maize, two DELLA proteins, DWARF8 (D8) and DWARF9 (D9), were identified [24], while the DELLA proteins REDUCED HEIGHT-1 (RHT-1) [25], SLENDER RICE1 (SLR1) [26], PROCERA [27], and VvGAI1 [28] were identified in wheat, rice, tomato, and grapevine, respectively. Additionally, four MeDELLAs have been identified in cassava [29].

DELLAs are important in plant development and growth and can interact with TCP transcript factors to affect the development of the inflorescence shoot apex and thus control plant height [29]. Leaf senescence can also be regulated by DELLAs [30]. DELLA proteins can regulate plant reproductive organ size, affect fertilization, and promote fruit growth [31]. Furthermore, DELLA proteins can promote the development of nodules and formation of infection threads during root nodule symbiosis; they can also control arbuscular mycorrhizal symbiosis in plants [32–34].

DELLAs play important roles in several aspects of plant development and growth that are influenced by environmental cues [35–40]. As such, DELLA proteins can improve the survival of plants during adversity [35, 36]. Researchers have concluded that DELLA proteins can integrate environmental signals, so that plants can alter their growth in response to the surrounding environment. DELLA proteins are also involved in responses to biotic stress [37, 41]. DELLA proteins can improve the tolerance of necrotrophs by potentiating jasmonate signaling [41]. Additionally, DELLA proteins can suppress laccase-like multicopper oxidase activity in response to herbivory and regulate glucosinolate levels [42].

DELLAs in many plant taxa have not yet been systematically analyzed. In this study, we searched for DELLA genes across 58 plant genomes and found a total of 181 DELLAs. This work provides helpful insights into the evolution of DELLA genes and offers important opportunities for the investigation of DELLA functions in different plant species, building upon previous research conducted on DELLA genes in many species. We performed a comprehensive analysis of DELLA genes throughout the plant kingdom, including analyses of their protein sequences, molecular signatures of selection, and codon usage patterns. We also analyzed the gene tree of these DELLAs using phylogenetic methods and identified orthologs, including PSGs, NSGs, and paralogs in 58 plant species. In addition, we performed other molecular evolution analyses of orthologs, paralogs, PSGs and NSGs, respectively, and relatively new and old DELLA genes, as well as improving knowledge about the VHYNP domain of DELLA proteins.

## Results

### Validation and extension of DELLA gene databases

Using the HMMER3 hmm search command, we identified 181 DELLAs from the 66 green plant genomes examined. Among the genomes of all Chlorophyta and the moss *Physcomitrella patens*, DELLA genes were not identified using the method described. Accordingly, DELLA genes were identified in 58 species in this study. To validate our searching method, we compared our list of DELLA genes with those reported in the literature. Our search successfully found all five DELLAs previously reported in *A. thaliana* [43], one reported in tomato [44], four reported in *Populus trichocarpa* [44], two reported in maize [24], one reported in rice [45], and two reported in *Selaginella moellendorffii* [29]. A previous study identified four DELLA genes in soybean [44], and two additional DELLA genes were identified in this species. Three DELLA genes were previously identified in the *Medicago truncatula* Mt3.5 genome database, and we only identified two DELLA genes in the newer *M. truncatula* Mt4.0 genome database (https://phytozome.jgi.doe.gov/pz/portal.html#!info?alias=Org_Mtruncatula). Furthermore, four DELLA genes were previously identified in cassava [46], while we only identified three DELLA genes in cassava based on a pfam domain hidden Markov model (HMM) database (Table S1).

Among bryophytes, we identified two DELLA genes in *Sphagnum fallax* and one in *Marchantia polymorpha*. In *Amborella trichopoda*, which belongs to the order Amborellales, we identified three DELLA genes. Among Gymnosperms, we identified two DELLA genes in *Ginkgo biloba*, two *in Pinus pinaster,* and one in *Picea abies*. Interestingly, in *Picea abies*, there was a protein with a sequence similar (identity > 80%) to that of a *Pinus pinaster* DELLA gene (sp_v3.0_unigene6469); however, the protein was not found by our identification strategy, since it does not contain a DELLA domain. Accordingly, it is not strictly considered to be a DELLA gene. We found that the apple genome examined contains the most DELLA genes, i.e., 14 in total. Most monocotyledons were found to contain only one DELLA gene. The longest DELLA sequence was that of GSVIVT01015465001 in grape, while the shortest was MDP0000855334 in apple (Table S1). The number of DELLA genes identified was neither correlated with the number of whole-genome duplications (WGDs) nor genome size, indicating that WGD events did not directly affect the number of DELLA genes.

**Fig. 1:**
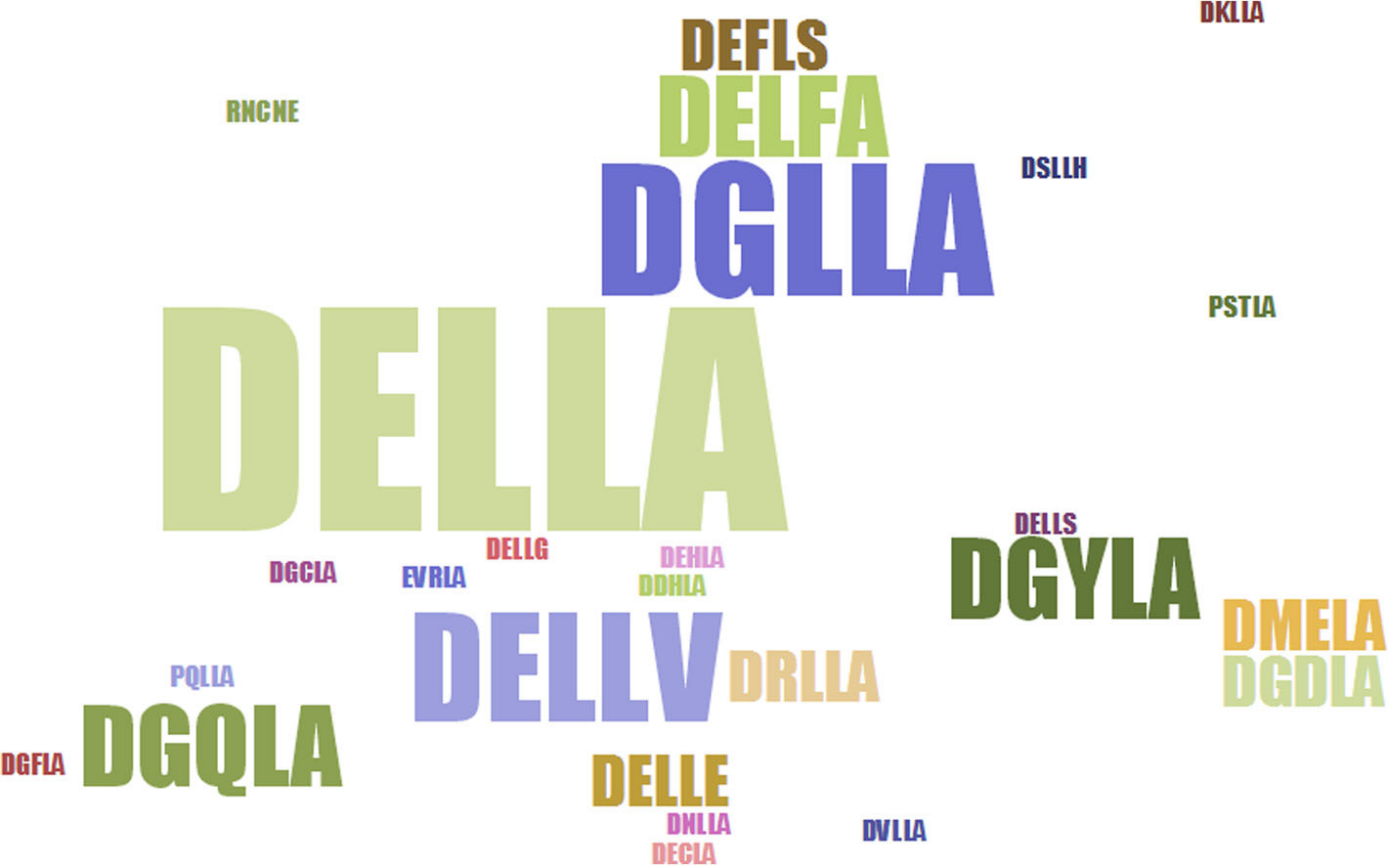
Different types of five-peptide “D-E-L-L-A” structures of plant DELLAs. The different sequences of letters represent the amino acid composition of different “D-E-L-L-A” structures. Larger characters show a larger proportion of this kind of structure

We also determined the conserved motifs and domains of these plant DELLA proteins. Using MEME, we identified 10 conserved motifs (Fig. S1). Motif7 partly overlaps with most DELLA domains, excluding Sphfalx0442s0002.1 in *Sphagnum fallax*, 122,441 in *Selaginella moellendorffii*, GSVIVT01030735001 in grapevine, MDP0000855334, MDP0000298557, MDP0000220512, and MDP0000192154 in apple, Spipo15G0028300 in *Spirodela polyrhiza*, and GSMUA_Achr9T13490_001 in *Musa acuminata* (Fig. S2). Most copies of motif7 started with “MDELLA” or “YDELLA” amino acid sequences. However, nine DELLA proteins do not contain motif7, and their DELLA domains differed more from other DELLA proteins and were shorter as well, such as apple DELLA protein MDP0000855334. Moreover, 67 DELLA domains do not contain “DELLA” sequences and instead contained domains, such as DGLLA, DSLLH, and other peptide strings of the “DXLLX” form. Additionally, some DELLA domains contain neither “DELLA” sequences nor the “DXLLX” form. For example, in *Sphagnum fallax*, DELLA Sphfalx0198s0025.1 does not contain a typical “DELLA” or “DXLLX” five-peptide structure, but instead contains an “RNCNE” structure (Fig. 1).

Most plant DELLA proteins contain GRAS domains. We found most DELLA proteins contain one DELLA domain and one GRAS domain. Some only contain a DELLA domain; some contain one DELLA domain and two GRAS domains; some contain one DELLA domain and three GRAS domains, and others contain two DELLA domains and one GRAS domain. Grape DELLA protein GSVIVT01015465001 contains one DELLA domain, one F-box domain, and one GRAS domain. Apple DELLA protein MDP0000220512 contains one DELLA domain and one WW domain. Apple DELLA protein MDP0000403162 contains one DELLA domain and one BCIP domain (Table S1).

**Fig. 2:**
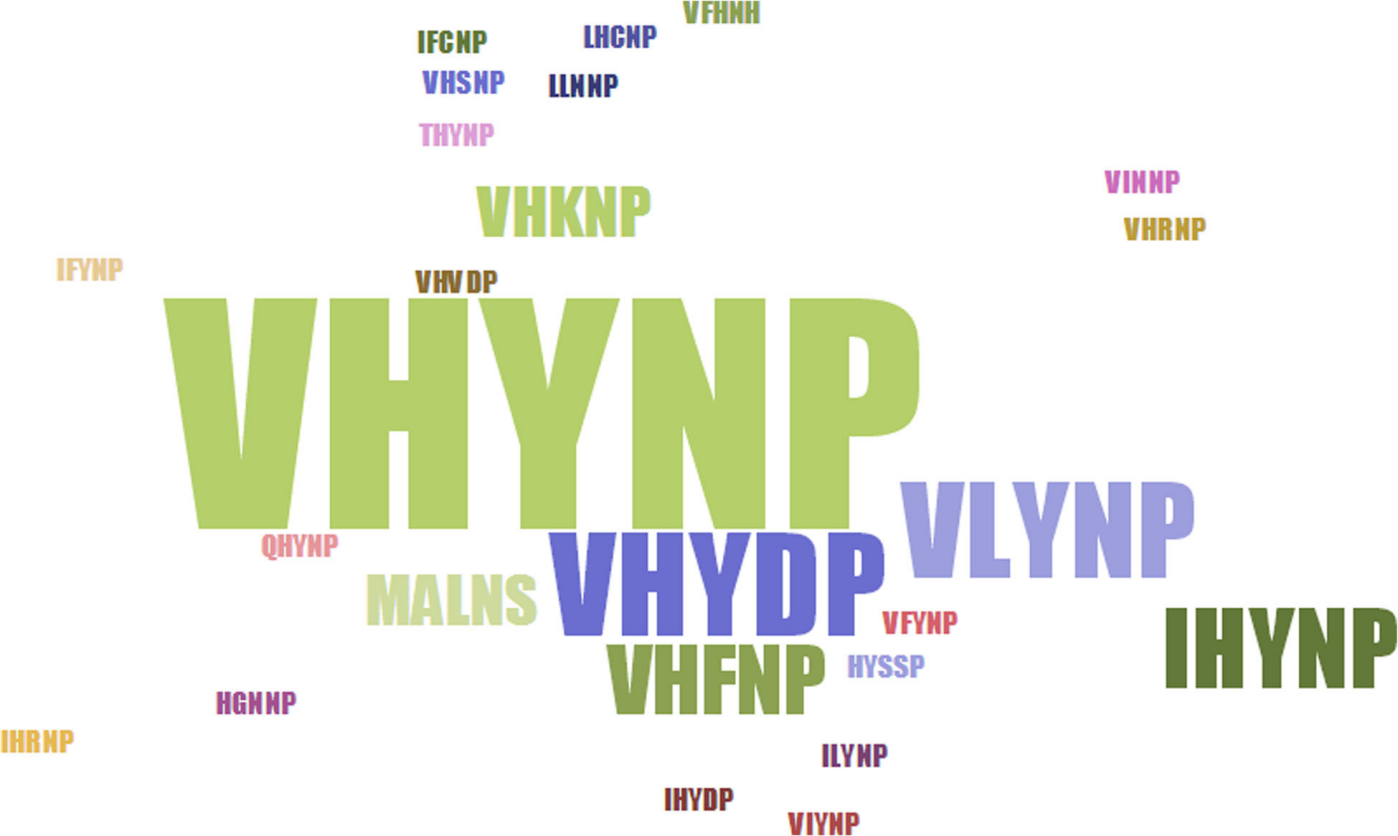
Different types of “VHYNP” sequences of plant DELLAs. The different sequences of letters represent amino acid composition of different “VHYNP” sequences. Larger characters show a larger proportion of this kind of sequence

The VHYNP motif of DELLA was identified based on its “VHYNP” sequences. VHYNP motifs were reported in rice as part of the “TVHYNP” domain [47]. VHYNP motifs of the DELLA protein RGA are needed for GID1 interactions in *A. thaliana* [48]. Previous studies have only reported VHYNP motifs in rice, maize, and *Arabidopsis* [48, 49], and Cassani et al. even referred to it as a putative VHYNP motif [49]. However, VHYNP motifs were not reported in other plants, and there was not a related pfam domain HMM in the pfam database. Motif10 partly overlaps with VHYNP motifs (Fig. S2). Because there is no VHYNP pfam domain HMM, we identified the VHYNP motifs of plant DELLA proteins based on identification of motif10 in plant DELLA proteins. Through detecting these instances of motif10, we found five DELLA proteins that did not contain a motif10/VHYNP motif. “VHYNP” motifs comprise 23 kinds of sequences, including “VHYNP,” “VHVDP,” and “VFYNP” sequences (Fig. 2).

### Gene tree analysis of DELLA sequences

**Fig. 3:**
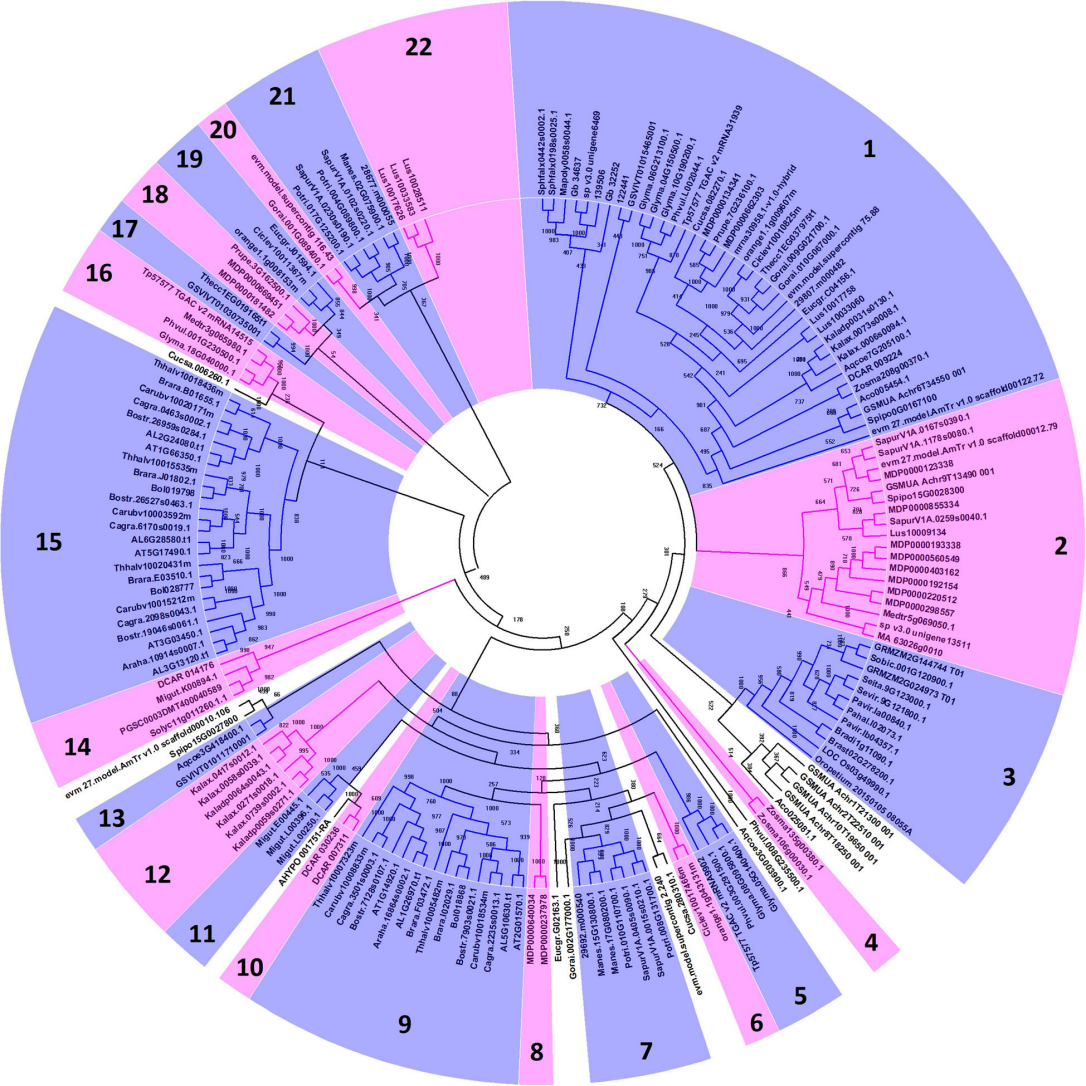
Evolution tree of plant DELLAS. The biggest numbers represent names of homologous groups. The numbers in branches represent the bootstrap values of branches

Because the ancestral DELLA gene sequence is unknown, and the extant gene closest to the ancestral gene sequence is unknown, we constructed an unrooted gene tree without an outgroup. Our gene tree analysis included all DELLA proteins identified in our search. We used the entire prepropeptide in our gene tree analysis. A previous study showed that plant DELLAs can be divided into three large groups. Here, our gene tree could be divided into 22 homologous groups based on bootstrap value (> 700), with some orphans, such as the *Amaranthus hypochondriacus* DELLA, AHYPO_001751-RA (Fig. 3). A previous study showed a group that contained genes from different species with a bootstrap value > 700 could be considered an orthologous group [50]. Here, paralogous groups only contained genes from the same species with a bootstrap value of at least 700 and could not be included in any other group with a bootstrap value of at least 700. Accordingly, the DELLAs from the same species in the same homologous group were considered paralogs, and the DELLAs from different species in the same homologous group were considered orthologs. In other words, if a homologous group contained DELLAs from different species, it could also be called an orthologous group. Based on bootstrap values, we identified 17 homologous groups, including groups 1, 2, 3, 5, 6, 7, 9, 12, and 13–21. (Fig. 3). Within an orthologous group, the genes included are orthologs of each other. Group 1 contained the most DELLAs and species (i.e., 30 species). DELLAs of the non-spermatophyte plants, i.e., bryophytes and *Selaginella*, only belonged to group 1. DELLAs from 10 monocotyledons (all gramineous species) are independently grouped into group 3. *Zostera marina* and *Spirodela polyrhiza* are aquatic angiosperms. One *Z. marina* DELLA (Zosma208g00370).1 was grouped into group 1, and two other *Z. marina* DELLAs were grouped into group 4. One *S. polyrhiza* DELLA (Spipo0G0167100) was grouped into group 1, while another *S. polyrhiza* DELLA (Spipo15G0028300) was grouped into group 2, and yet another (Spipo15G0027800) was an orphan. DELLAs of nine types of cruciferous plants, including *Arabidopsis*, were independently divided into groups 9 and 15. The result is in agreement with a previous study, in which *Arabidopsis* DELLA GAI (AT1G14920.1) and RGA (AT2G01570.1) were grouped into group 9, while RGL1 (AT1G66350.1), RGL2 (AT3G03450.1), and RGL3 (AT5G17490.1) were grouped into group 15 [44]. Cruciferous *Arabidopsis thaliana*, *Capsella grandiflora*, *Boechera stricta*, *Arabidopsis lyrata,* and *Capsella rubella* all contain five DELLAs, and all have two DELLAs in group 9 and three DELLAs in group 15. However, *A. halleri* only contained two DELLAs, one in group 9 and one in group 15. Group 16 only contains DELLAs of leguminous plants. Group 22 only contains the DELLAs of *Linum usitatissimum*. Most apple DELLAs were divided into groups 1 and 2, and group 12 only contained *Kalanchoe* DELLAs (Fig. 3).

Paralogs were derived from duplication events. Non-paralogous (non-duplication) DELLA genes were considered singleton DELLA genes in our study. Accordingly, we identified 101 duplicate DELLA genes. Grape DELLA genes were all singletons, while all apple DELLA genes were duplicates. Most soybean DELLA genes were duplicates, but one was a singleton.

### Molecular evolution analysis of orthologous groups, PSGs, NSGs, and new and old DELLA genes

For each orthologous group, *K*_a_, *K*_s_, and *K*_a_/*K*_s_ (ω) values were calculated (Table 1), where *K*_a_ and *K*_s_ represent rates of molecular evolution. Overall, *K*_a_ ranged from 0.0122 to 8.8684, with a mean of 1.48 (Table 1). The *K*_s_ estimates ranged from 0.0164 in group 6 to 12.8 in group 1, with a mean of 3.160 (Table 1). These results showed that the divergence time of group 1 was most ancient, while that of group 6 was the most recent. The *K*_a_/*K*_s_ estimates ranged from 0.0389 to 3.966, with a mean of 0.748 (Table 1). Previous studies have shown that genes could be classified into PSGs and NSGs, which have undergone purifying selection and neutral genes [51]. The identification of PSGs and NSGs is based on the separate *K*_a_/*K*_s_ values of each orthologous group. If the ω vales of an orthologous group exceed 1, the genes are considered PSGs [52, 53]. If the ω value of an orthologous group is less than 1, the genes are considered NSGs [52, 53]. In our study, ω values of four orthologous groups were found to be greater than 1, while the others were less than 1. In this study, 35 PSGs and 121 NSGs were found among all the DELLA genes (Table S2). We found that *K*_a_/*K*_s_ increases gradually as *K*_s_ increases in the NSGs (*r* = 0.5). *K*_a_/*K*_s_ was negatively correlated with *K*_s_ (*r* = − 0.65) among the PSGs. As shown in Table 2, the average *K*_s_ of NSGs is 3.9, which was significantly higher than the average *K*_s_ of PSGs (0.72; *P* < 0.05). We also calculated the codon preference index and found that the average codon adaption index (CAI) and GC and GC3 contents of NSGs were significantly higher than those of PSGs (Table 2).

**Table 1:** Ka, Ks and Ka/Ks value of all homologous groups

Group name	Ka	Ks	Ka/Ks
Group1	8.8684	12.8116	0.69222
Group2	8.375	9.9533	0.84143
Group3	0.2495	2.8828	0.08654
Group5	0.966	0.4598	2.10117
Group6	0.0122	0.0164	0.74373
Group7	0.3385	2.1128	0.16021
Group9	0.6163	3.6567	0.16855
Group12	0.0879	0.504	0.17445
Group13	0.7823	0.4619	1.69379
Group14	0.4106	6.8241	0.06017
Group15	2.6585	1.8314	1.4516
Group16	0.3155	2.4392	0.12935
Group17	0.3331	3.0723	0.10842
Group18	0.1154	0.775	0.14895
Group19	0.6071	0.1531	3.96623
Group20	0.1427	3.6662	0.03891
Group21	0.3323	2.2607	0.14701

**Table 2:** Various indexes of PSG and NSG

	NSG	PSG	
**Ks**	3.921 ± 3.625	0.726 ± 0.65	*
**Ka**	1.553 ± 0.766	1.253 ± 0.821	
**CAI**	0.238 ± 0.03	0.22 ± 0.02	*
**CBI**	0.1 ± 0.09	0.08 ± 0.05	
**Fop**	0.476 ± 0.05	0.477 ± 0.03	
**Exon length**	1533 ± 428	1620 ± 126	
**GC**	0.546 ± 0.077	0.515 ± 0.047	*
**GC3**	0.60 ± 0.17	50.4 ± 0.11	*

“*” represents significant difference (P < 0.05)

A previous study determined that the genes in a genome differ in age in the sense that they have identifiable orthologs across a diverse range of species spanning vast evolutionary distances. Some genes are younger, in the sense that orthologs are identifiable only in closely related species [54]. Accordingly, we considered genes in orthologous groups to be old DELLA genes, while the others were considered new DELLA genes. The new genes included two DELLA genes in *Z. marina*, three in *Mimulus guttatus*, two in apple, two in *Daucus carota*, and three in *Linum usitatissimum*. The Fop, CAI, and CBI values of both new and old DELLA genes were positively correlated with GC content. The CAI values of new DELLA genes were positively correlated with exon length. CBI and Fop of old DELLA genes were positively correlated with exon length (*r* > 0.1). The GC12 content of both new and old DELLA genes was significantly positively correlated with GC3 content (*r* > 0.75, *P* < 0.01; Table 3).

**Table 3:** Correlation analysis among various indexes of new and old genes

New gene			
	CAI	CBI	Fop
**GC**	0.27403	0.61746*	0.59282*
**exon length**	0.10988	−0.02117	0.00402
**GC1**	0.1788	0.47283*	0.43599*
**GC2**	0.1375	0.47316*	0.46518*
**GC12**	0.16778	0.49214*	0.46658*
**GC3**	0.32925	0.66561*	0.6429*
**Old gene**			
	**CAI**	**CBI**	**Fop**
**GC**	0.49037*	0.78934*	0.77408*
**exon length**	0.04337	0.16199*	0.15033
**GC1**	0.465*	0.66137*	0.63796*
**GC2**	0.18153*	0.36352*	0.36707*
**GC12**	0.38737*	0.60739*	0.59468*
**GC3**	0.49707*	0.80981*	0.79306*

“*” represents significant correlation (P < 0.05)

### Identification and molecular evolution analysis of paralogous groups

A previous study considered a group containing genes from the same species with a bootstrap value > 700 to be a paralogous group within a gene tree [50], regardless of whether or not the group is already included in an orthologous group. In group 1, we identified nine paralogous groups, including a positively selected paralogous group of *Selaginella moellendorffii* DELLA genes. In group 2, we identified two paralogous groups, including a positively selected paralogous group of apple DELLA genes. In homologous group 3, we identified two paralogous groups. Group 4 is also a paralogous group that only includes two *Z. marina* DELLA genes. In group 5, we identified one paralogous group, including two soybean DELLA genes. In group 7, we identified one paralogous group, including two *Manihot esculenta* DELLA genes. Group 8 is also a paralogous group that only includes two apple DELLA genes. In group 9, we identified seven paralogous groups, including a positively selected paralogous group of *Arabidopsis lyrate* DELLA genes. Groups 10 and 11 are also paralogous groups. In group 12, we identified two paralogous groups. In group 15, we identified eight paralogous groups, including a positive paralogous group of *A. lyrate* DELLA genes. In group 21, we identified two paralogous groups, and in orthologous group 22, we identified one paralogous group. Group 22 is also a paralogous group consisting of only three apple genes (Fig. 3).

We also compared the average *K*_a_, *K*_s_, and ω values of paralogous groups with those of orthologous groups. The average ω value of paralogous groups was 0.46, and the average ω value of orthologous groups was 0.7. The average *K*_s_ value of paralogous groups was 0.8, and the average *K*_s_ value of orthologous groups was 3.16. The average *K*_a_ value of paralogous groups was 0.36, and the average *K*_a_ of orthologous groups was 1.48. In both the orthologous and paralogous groups, the *K*_s_ values were negatively correlated with ω values, while the ω values were negatively correlated with *K*_s_ values and positively correlated with *K*_a_ values both in orthologous groups and paralogous groups (*r* > 0.1). In the paralogous groups, CBI and Fop were significantly positively correlated with GC, GC1, GC2, GC12, and GC3, and CAI was significantly positively correlated with GC, GC1, GC12, and GC (Table 4). The paralogous groups for which ω was greater than 1 had significantly higher average *K*_a_ values. Accordingly, they had faster rates of amino acid substitution and evolution. We also analyzed the motifs of paralogous groups for which ω was greater than 1. We found some paralogous groups for which ω was greater than 1 that differed with respect to their motifs; for example, one group, containing DELLAs 122,441 and 139,506, with the latter containing motif7. However, 122,441 did not contain motif7 (Fig. S1); this may have been caused by positive selection leading to a new gene function [55].

**Table 4:** Correlation analysis among various indexes of paralogous

	CAI	CBI	Fop
**GC**	0.52825*	0.76662*	0.75238*
**GC1**	0.42804*	0.53809*	0.50243*
**GC2**	0.10389	0.26008*	0.26722*
**GC12**	0.33572*	0.50442*	0.4865*
**GC3**	0.55326*	0.79438*	0.78327*
**exon length**	−0.00552	0.14136	0.12601*

“*” represents significant correlation (P < 0.05)

## Discussion

We identified DELLA genes throughout the plant kingdom but were unable to find DELLA genes from green algae, demonstrating that DELLAs were derived in the lineage leading to land plants. Previous studies have failed to report DELLAs in bryophyte genomes; however, we found DELLAs in *Marchantia polymorpha* and *Sphagnum fallax*, although we did not find DELLAs in *Physcomitrella patens*. Accordingly, it is unclear which bryophytes contain DELLA genes, and this remains to be studied in further detail. Chlorophyta, which live in water, do not have DELLAs. However, *Z. marina* and *S. polyrhiza*, which are aquatic vascular plants, do have DELLAs. *Z. marina* and *S. polyrhiza* are angiosperms, which demonstrate that aquatic vascular plants have not lost DELLAs.

The expansion of many other reported gene families has also been related to WGD events [46]. We found that the number of DELLA genes was not related to genome size or the number of WGD events. Accordingly, we deduced that WGD events have not led to the expansion of the DELLA gene family. Gene family contraction can also be related to gene loss events [56]. In *Picea abies*, there is a protein that is highly similar (identity > 80%) to a DELLA gene in *Pinus pinaster* (sp_v3.0_unigene6469); however, the protein was not identified by our identification strategy. Because it does not contain a DELLA domain, it is not considered to be a DELLA gene. This may therefore be considered a loss event of a DELLA gene in *Picea abies*. Similarly, most cruciferous plants contained five DELLA genes, but a few contained two DELLA genes, which may also be explained by one or more loss events.

**Fig. 4:**
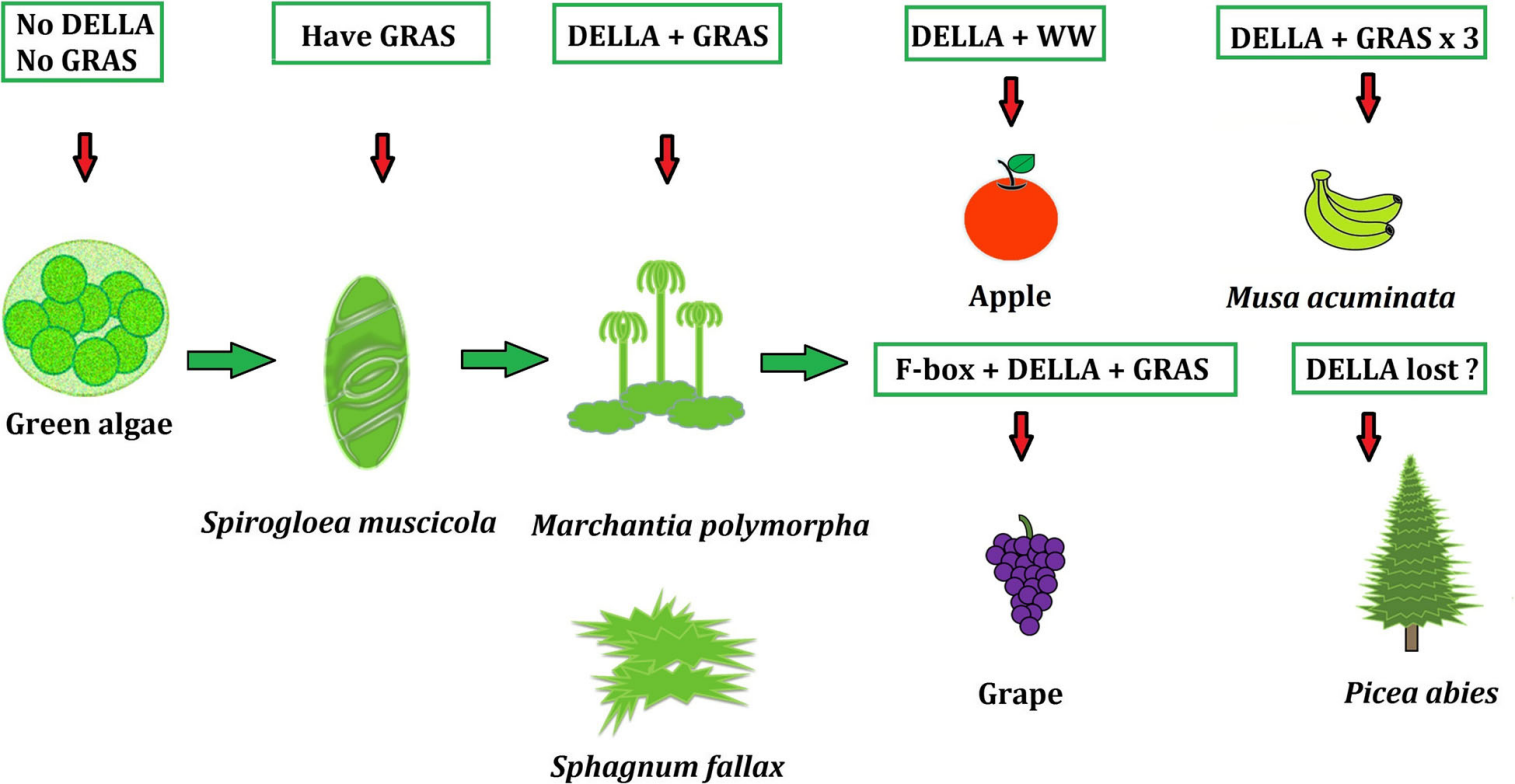
DELLA domain and other fused domains in plant evolution. During the process of plant evolution, some genomes acquired DELLA and GRAS domains. Neither DELLA nor GRAS domains are found in some green algae, although there are GRAS domains in *Spirogloea muscicola*. Similarly, there are DELLA domains and GRAS domains in some mosses. The WW domain is integrated into an apple DELLA, but its GRAS domain is missing. A fusion of the F-box domain is found in a grape DELLA. In wild banana, the number of GRAS domains is tripled. A loss event of a DELLA domain appears to have happened in *Picea abies*

A previous study strictly described the “D-E-L-L-A” structure of the five-peptide ELLA domain [57]. However, we found many related five-peptide structures that had diversified and were not “DELLA” specifically, including “DGLLA,” “DSLLH,” and other peptide strings of the “DXLLX” form. Additionally, some DELLA domains contain neither “DELLA” sequences nor sequences in the “DXLLX” form. This shows the variability of DELLA domain structure. In the fern *Selaginella*, one DELLA gene had the five-peptide “D-E-L-L-A” structure. Similarly, most terrestrial monocotyledons contain DELLA genes with a five-peptide “D-E-L-L-A” structure. *Z. marina*, an aquatic monocotyledon, has DELLA genes that do not contain the typical five-peptide “D-E-L-L-A” structure. A previous study reported that the DELLA family is a subfamily of the GRAS superfamily [58]. However, we found that many plant DELLAs do not contain the GRAS domain, such as the apple DELLAs MDP0000192154 and MDP0000220512. A previous study reported that early plants (e.g., *Spirogloea muscicola*) gained a GRAS domain from microorganisms in the soil [59]. While the origins of the DELLA domain remain unknown, we were able to find DELLA domains in some bryophytes. Previous studies have also reported that the DELLA is a type of GRAS protein, because those reported DELLAs were found to contain GRAS domains. Here, we found some DELLAs without GRAS domains. This raises the question of whether a DELLA protein is a GRAS protein. Here, we found one DELLA that also contained a fused F-box domain (grape DELLA, GSVIVT01015465001), and one that contained a fused WW domain (apple DELLA, MDP0000220512). Some *Musa acuminate* DELLAs contained three repeat GRAS domains, and it appears that a DELLA domain loss event happened in *Picea abies* (Fig. 4). A previous study also reported some details about the VHYNP motif in DELLA genes [57]. Here, we found most plant DELLAs contain the VHYNP motif, and these related motifs are also diverse, including five-peptide “VHYNP,” “VHVDP,” and “VFYNP” structures.

Our gene tree analysis revealed results that contrasted with a previous study that divided DELLAs into three groups based on approximately one dozen species [44]. Here, we divided DELLAs into 22 groups, with some orphan groups, based on many more species. In other words, we found as many as 22 orthologous groups. Orthologs often share similar functions [60]. Accordingly, we conclude that DELLA genes may have become very functionally diversified throughout the plant kingdom. Accordingly, many functions of the plant DELLA genes remain unknown. A previous study reported that DELLA genes from cruciferous plants could be divided into two groups, which is consistent with the results of our study. However, our finding is that DELLA genes from crucifers could be divided into two independent groups without any other plants. We found that group 1 contains most types of plants, other than those in the families Cruciferae and Gramineae. The Gramineae DELLA genes could be divided into an independent group. Only group 1 contained bryophyte DELLA genes, showing that group 1 may correspond to the ancestral gene family members. Additionally, one aquatic *Z. marina* DELLA was assigned to group 1, showing that this gene copy may be derived from the ancestral copy of the many other DELLAs.

Among these orthologous groups, some PSGs were also identified. PSGs were deduced to be under positive selection throughout evolutionary history, with positive selection being related to the adaptation to environments of these species [61–63]. In the plant kingdom, many DELLA genes appear to be involved in tolerance of various abiotic stresses and a range of developmental processes [29–32, 35, 36]. Orthologous groups contain PSGs that may exhibit functional divergence or the evolution of new DELLA gene functions [55, 61–63]. Accordingly, the evolution of plant DELLA genes appears to have been closely related to environmental adaptation and appears to have driven diverse functions underlying adaptation to the environment. Two *Z. marina* DELLA genes were independently included into one orthologous group (and were also considered to be two new genes); accordingly, the two DELLA genes may pertain to adaptation to aquatic environments. This suggests that various plant DELLAs may still have many unknown functions awaiting discovery. We also found the molecular evolutionary rate of DELLA orthologs to be faster than that of paralogs. Accordingly, the emergence of new species is likely to be associated with new functions among DELLA genes. Species diversification may be a driving force for the emergence of DELLA genes with different functions or multiple functions, as adaptation differs among species under different environments or with different developmental patterns.

The average *K*_s_ values of NSGs were significantly higher than those of PSGs, indicating that the average molecular evolutionary rate of DELLA NSGs was higher [64]. The average *K*_s_ of PSGs being significantly lower also shows that the average divergence time of orthologous PSGs occurred later [50, 53, 61, 65]. This also shows that the DELLA family may have primarily undergone negative selection at first, with positive selection occurring later. PSGs among the orthologous groups were under positive selection. Previous studies have shown that positive selection may be involved in gene function losses, adaptive evolution, and pseudogenization [62–64]. Positive selection could also lead to new gene functions [55]. For example, homologous group 13 is an orthologous group that contains PSGs, with some PSGs in group 13 containing different motifs. Moreover, different motifs may be related to different functions; in other words, the functions of some PSGs may be different between orthologs. Accordingly, we presumed that recent divergence (sequence divergence or nucleotide substitution) may be more related to the function divergence of DELLAs.

Within the paralogous groups, CBI and Fop were significantly positively correlated with GC3, GC2, GC1, GC, and GC12. Within paralogous groups, CAI was also significantly positively correlated with GC12, GC, GC1, and GC. This showed that paralogs underwent mutation selection [66, 67]. The paralogous groups for which ω exceeds 1 have significantly higher *K*_a_ values, indicating that their rate of amino acid substitution is higher. Some paralogous groups for which ω exceeds 1 differed in their motifs, and different motifs may be related to different functions. This shows that positive selection may have led to new functions of DELLA genes [55]. In future work, we aspire to determine whether PSGs and paralogs with different motifs have functions that differ from their homologs based on the results of this study. We could also detect whether DELLAs with specific domains correspond to specific functions, and this work is poised to discover additional new functions of DELLA proteins.

## Conclusions

We searched for DELLA genes across 58 green plant genomes and found a total of 181 DELLAs. We analyzed the gene tree of these DELLAs using phylogenetic methods and identified orthologs and paralogs in 58 plant species. We also performed molecular evolution analyses of PSGs, NSGs, and new and old DELLA genes, respectively, as well as improving the knowledge about VHYNP domain of DELLA proteins. Our study provides helpful insights into the evolution of DELLA genes and offers substantial opportunities for the investigation of DELLA functions across plant species.

## Methods

### Data collection and identification of plant DELLA genes

In total, 66 plant genomes were obtained for analysis, including 63 green plant genomes from the Phytozome 12.1.6 database (https://phytozome.jgi.doe.gov/pz/portal.html) and the gymnosperm genomes of *Ginkgo biloba* [68] from GigaDB (http://gigadb.org/), *Pinus pinaster* from SustainPine DB (http://www.scbi.uma.es/pindb/), and *Picea abies* from the Congenie.org database (http://congenie.org). Based on the above data, we used the HMMER3 hmm search command to identify all possible DELLA protein candidates in the plant genome database [69]. We used the online software SMART (http://smart.embl-heidelberg.de/) to identify integrated DELLA domains within putative plant DELLA proteins [70].

### Phylogenetic analysis of plant DELLA proteins

Plant DELLA protein sequence alignment was performed using ClustalX software Version 2.1 [71] followed by the PHYLIP software package [72] to construct neighbor-joining (NJ) trees from the ClustalX alignment of the DELLAs. 1000 bootstrap replicates were generated to estimate support for the inferred relationships [73].

### Conserved motif analysis of plant DELLAs

Conserved motifs in plant DELLAs were analyzed using the software MEME suite (version 4.11.1; http://meme.nbcr.net/meme/) [74]. Parameter settings: minimum motif width, 6; output motifs, 20; maximum motif width, 300 [73].

### Selective pressure analysis

The ratios of non-synonymous to synonymous substitutions (i.e., *K*_a_/*K*_s_ or ω) of gene groups were calculated using Codeml as implemented in PAML version 4.7 software [75].

### Codon usage bias analysis

Parameters reflecting codon usage bias, including FOP, GC1, GC2, GC3s, GC12 content, RSCU, CAI, CBI, and exon length, were calculated using CodonW version 1.4.2 [76].

## Supplementary information

**Additional file 1: Table S1.** Amino acid sequences of DELLAs in various species. Information includes the species name, gene name, amino acid sequence, and domain composition.

**Additional file 2: Table S2.** CDs sequences of DELLA NSGs and PSGs.

**Additional file 3: Fig. S1.** Conserved motifs of plant DELLAs.

**Additional file 4: Fig. S2.** Conserved motif sequences of plant DELLAs. The red box contains the conserved amino acid sequence of five-peptide “D-E-L-L-A” structure. The green box contains the conserved amino acid sequence of the “VHYNP” sequence.

## Data Availability

66 plant genomes and related data were downloaded from the Phytozome 12.1.6 database (https://phytozome.jgi.doe.gov/pz/portal.html), and the gymnosperm genomes and related data of *Ginkgo biloba* [68] was downloaded from GigaDB (http://gigadb.org/), *Pinus pinaster* was downloaded from SustainPine DB (http://www.scbi.uma.es/pindb/), and *Picea abies* was downloaded from the Congenie.org database (http://congenie.org).

## Abbreviations

ABI: ABA INSENSITIVE
ARF: AUXIN RESPONSE FACTOR
BR: Brassinosteroid
PIF: PHYTOCHROME INTERACTING FACTOR
BZR1: BRASSINAZOLE-RESISTANT 1
EIN: ETHYLENE-INSENSITIVE
JAZ: JASMONATE-ZIM-DOMAIN PROTEIN
D14: DWARF 14
FLC: FLOWERING LOCUS C
RGL: RGALIKE
GAI: GA INSENSITIVE
SLR1: SLENDER RICE 1
JA: Jasmonate
PSG: Positively selected gene
NSG: Negatively selected gene
