# Supplementary figures and images for: Comprehensive identification and analysis of DELLA genes throughout the plant kingdom
Source: BMC Plant Biol. 2020 Aug 6;20:372. doi: 10.1186/s12870-020-02574-2 (PMC7409643; doi:10.1186/s12870-020-02574-2)

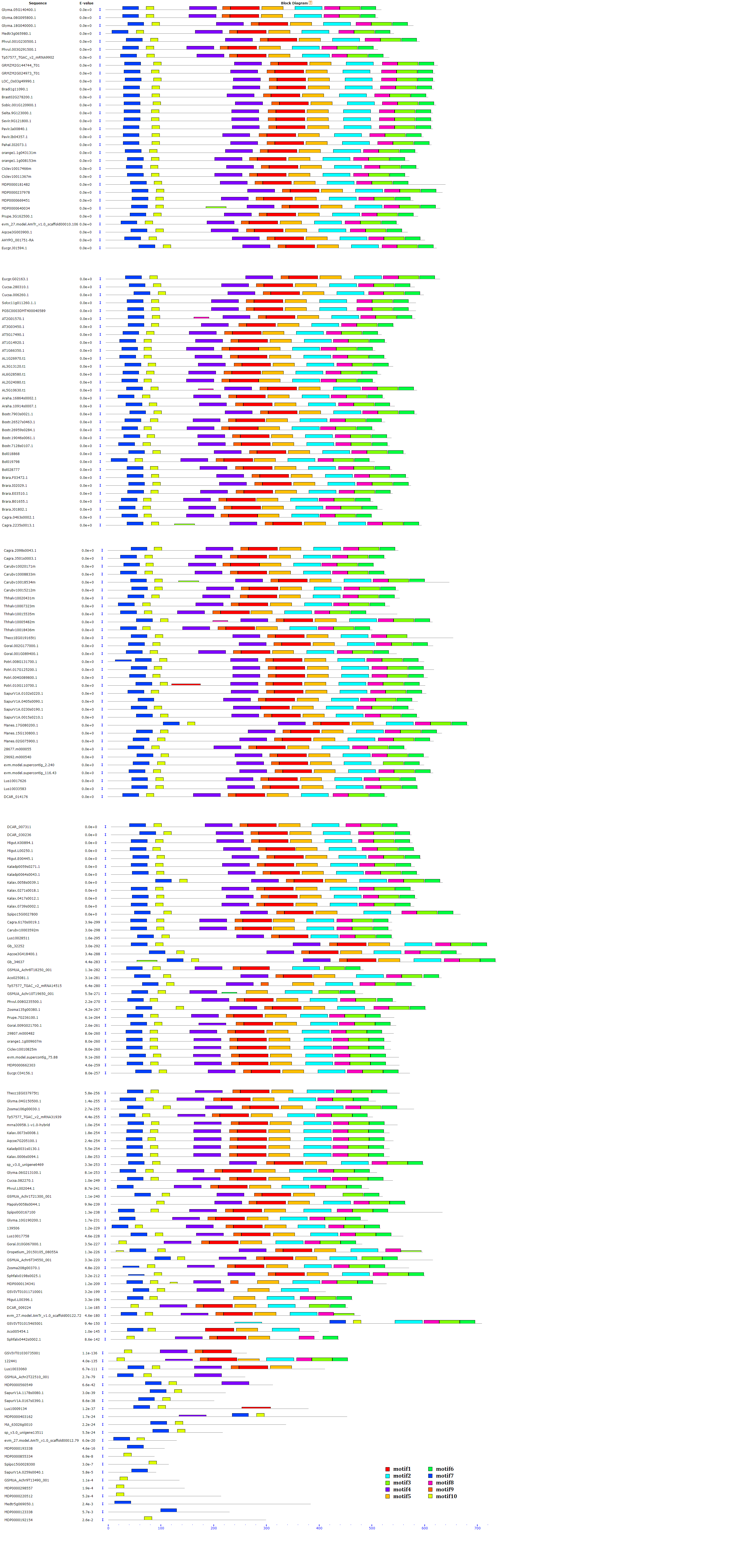

Supplement: Supplementary file 3 — Additional file 3: Fig. S1. Conserved motifs of plant DELLAs. [file 12870_2020_2574_MOESM3_ESM.png]

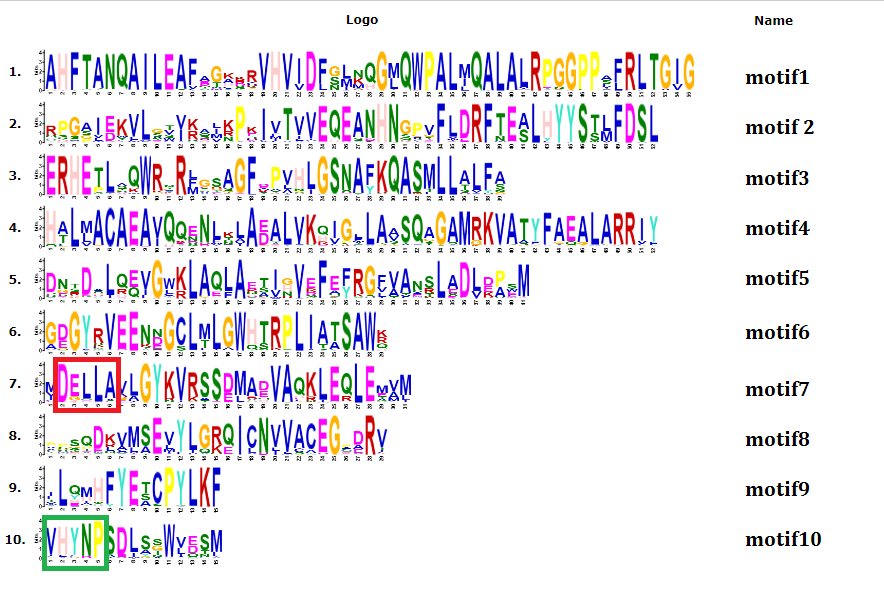

Supplement: Supplementary file 4 — Additional file 4: Fig. S2. Conserved motif sequences of plant DELLAs. The red box contains the conserved amino acid sequence of five-peptide “D-E-L-L-A” structure. The green box contains the conserved amino acid sequence of the “VHYNP” sequence. [file 12870_2020_2574_MOESM4_ESM.png]
